# Inpatient child mortality by travel time to hospital in a rural area of Tanzania

**DOI:** 10.1111/tmi.12294

**Published:** 2014-03-24

**Authors:** Rachel Manongi, Frank Mtei, George Mtove, Behzad Nadjm, Florida Muro, Victor Alegana, Abdisalan M. Noor, Jim Todd, Hugh Reyburn

**Affiliations:** 1 Kilimanjaro Christian Medical Centre, Moshi, Tanzania; 2 National Institute for Medical Research, Amani Centre, Muheza, Tanzania; 3 London School of Hygiene & Tropical Medicine, London, UK; 4 Oxford University Clinical Research Unit, Hanoi, Vietnam; 5 Malaria Public Health Department, Kenya Medical Research Institute-Wellcome Trust Research Programme, Nairobi, Kenya; 6 Centre for Tropical Medicine, Nuffield Department of Clinical Medicine, University of Oxford, Oxford, UK

**Keywords:** access, hospital, mortality, child, Africa

## Abstract

**OBJECTIVE:**

To investigate the association, if any, between child mortality and distance to the nearest hospital.

**METHODS:**

The study was based on data from a 1-year study of the cause of illness in febrile paediatric admissions to a district hospital in north-east Tanzania. All villages in the catchment population were geolocated, and travel times were estimated from availability of local transport. Using bands of travel time to hospital, we compared admission rates, inpatient case fatality rates and child mortality rates in the catchment population using inpatient deaths as the numerator.

**RESULTS:**

Three thousand hundred and eleven children under the age of 5 years were included of whom 4.6% died; 2307 were admitted from <3 h away of whom 3.4% died and 804 were admitted from ≥3 h away of whom 8.0% died. The admission rate declined from 125/1000 catchment population at <3 h away to 25/1000 at ≥3 h away, and the corresponding hospital deaths/catchment population were 4.3/1000 and 2.0/1000, respectively. Children admitted from more than 3 h away were more likely to be male, had a longer pre-admission duration of illness and a shorter time between admission and death. Assuming uniform mortality in the catchment population, the predicted number of deaths not benefiting from hospital admission prior to death increased by 21.4% per hour of travel time to hospital. If the same admission and death rates that were found at <3 h from the hospital applied to the whole catchment population and if hospital care conferred a 30% survival benefit compared to home care, then 10.3% of childhood deaths due to febrile illness in the catchment population would have been averted.

**CONCLUSIONS:**

The mortality impact of poor access to hospital care in areas of high paediatric mortality is likely to be substantial although uncertainty over the mortality benefit of inpatient care is the largest constraint in making an accurate estimate.

## Introduction

Treatment for children with acute febrile illness is one of a small number of basic and affordable interventions that have the potential to substantially reduce child mortality in resource-poor countries (Jones *et al.* 2003). Several studies have demonstrated a relationship between child mortality and physical access to the nearest primary care facility (Frankenberg 1995; van den Broeck *et al.* 1996; Magnani *et al.* 1996; Sankoh *et al.* 2001; Kadobera *et al.* 2012) although this may not apply where distances are relatively short (Rutherford *et al.* 2009, 2010; Becher *et al.* 2004). However, there are very few studies of the association between child mortality and access to hospital care for sick children in Africa where both travel times and facility-based mortality are much higher than those found among users of primary care services (Snow *et al.* 1994a,b).

Although access is a multidimensional concept, most African studies have focussed on geographical distance as a proxy measure for physical access. Studies from the Kenyan coast and from the Gambia have both documented a marked decline in hospital admission rates for common childhood conditions with increasing distance from the nearest main hospital. (Snow *et al.* 1994a,b; Schellenberg *et al.* 1998; Weber *et al.* 2002). However, to our knowledge, no previous study has estimated how this difference in use of hospital services for severely ill children is likely to impact on child mortality in the catchment population. Such knowledge could provide more accurate estimates to inform decisions on the use of scarce resources to improve access to inpatient services serving rural communities in Africa.

We have addressed this question by studying travel times and mortality among children admitted to a district hospital in north-east Tanzania over 1 year. Using the hospital catchment population, we have estimated the number of deaths that did not benefit from inpatient care as a result of poor physical access. While such an estimate is inevitably prone to a number of uncertainties, the results should stimulate a greater awareness of the importance of access to hospital for sick children when planning new roads or bus services or the location of inpatient facilities.

## Methods

### Overview

This is a secondary analysis of a 1-year observational study conducted in 2005–6 of paediatric admissions to Teule Hospital in north-east Tanzania, a district hospital serving a predominantly rural population with under 5 child mortality of 165/1000 (Nadjm *et al.* 2010). At the time of the study, *Plasmodium falciparum* transmission was intense (50–700 infected bites/person/year) and perennial. There is a single main road passing through the district, and remaining roads are mostly unpaved. A variety of bus and truck services run from villages to the district capital (Muheza) where the hospital is located. At the time of the study, there were few motorcycle services. Bicycle use is common but is not commonly used to transport sick children.

### Eligibility and enrolment

Consecutive daytime paediatric admissions were provided with emergency treatment if needed and then screened for study eligibility. Inclusion criteria were age 2 months to 13 years with a current fever or history of fever within the previous 48 h. In this analysis, only children aged 2–59 months (91% of the total children in the study) were included to conform to national census age categories. Children with an obvious non-infectious cause for admission such as trauma, surgery or known malignancy were excluded. Children were enrolled over five consecutive days each week. To include children presenting outside of normal working hours, children were recruited from Monday to Friday for the first 7 months and Wednesday to Sunday for the second 5 months.

### Clinical and laboratory data collection

Following consenting procedures, children were assessed by clinical officers (a grade of non-physician clinician in Tanzania) using a standard medical history and examination. Clinical data collection was supervised throughout the study by two experienced physicians (BN & GM). A study of interobserver variation of clinical signs was conducted during the study, partly undertaken as quality control measure (Nadjm *et al.* 2008). Blood from each child in the study was tested for blood stream bacterial infection by aerobic blood culture, presence of *P. falciparum* asexual parasitaemia and HIV serology. All laboratory tests were undertaken according to rigorous research standards as described elsewhere. Case management was consistent with WHO guidelines with the addition that all children with severe malaria were treated with broad-spectrum antibiotics. Admission outcomes were recorded at discharge or death.

### Census and mapping data and estimated catchment population

National census data were used for population estimates (National Bureau of Statistics, United Republic of Tanzania 2002) (Anyangu *et al.* 2010). The spatial positions of the villages, hospitals, main and peripheral roads and primary care facilities in Muheza district and neighbouring areas were assembled in ArcGIS version 9.3 (ESRI, Inc., Redland, CA, USA). Linear distances were adjusted for the presence of rivers, hills or other impediments between each village and Teule hospital l using the AccessMod package in Arcview 3.3 (ESRI, Inc.). (Alegana *et al.* 2012).

**Figure 1:**
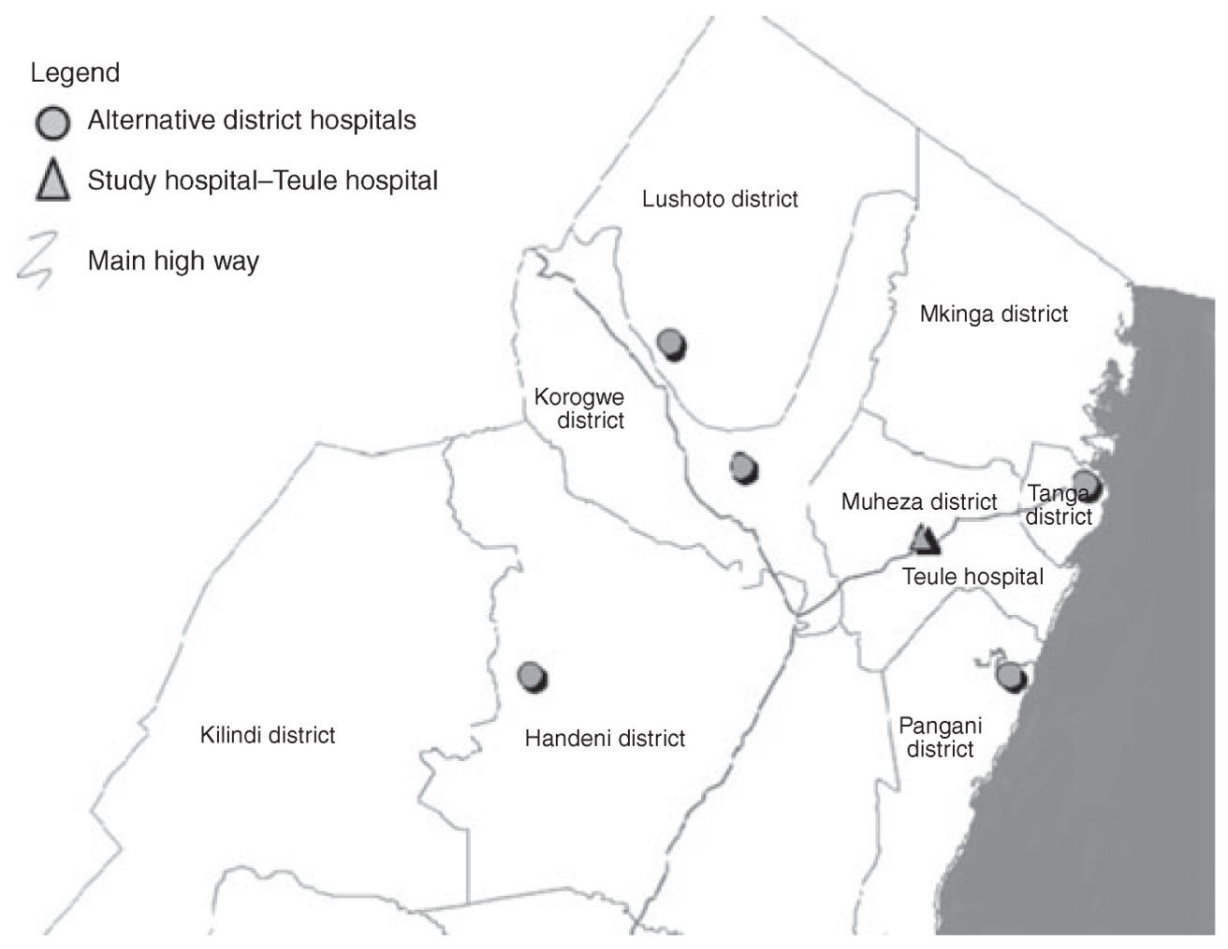
Sketch map of the study area.

The spatial positions of the hospitals within the region were assembled, and the catchment population was defined as the population of villages that were nearest (by distance adjusted as above) to Teule Hospital, a methodology consistent with other studies (Okiro *et al.* 2009). This assumes that all hospitals had an equally weighted draw on patients and local enquiry suggested that this was true (Figure 1).

Travel time was calculated using criteria based on reports by local residents of how they would normally undertake the journey to Teule hospital; those living at <7 km adjusted distance were assumed to walk at 3 km/h, those living >7 km from the hospital but within 5 km of a main road were assumed to travel at 4 km/h to the main road, to wait for 30 min for a bus, then to travel at 30 km/h to the district town and finally to walk 20 min to the hospital itself. Those living more than 7 km from the hospital and 10 km or more from the main road were assumed to use some form of motorised transport for a part of the journey and thus to travel at an average speed of 8 km/h to the main road following which travel times would be the same as for those specified above.

### Data management and analysis

Data were scanned using the Cardiff Teleform system (Cardiff Inc. Vista, CA., USA) into an Access database (Microsoft Corp, Va., USA) and analysed in Stata-10 (Stata Corp, TX, USA). Nutritional *Z*-scores were calculated from NCHS/WHO reference data in Epi-6 (CDC, Atlanta, USA). Poisson’s regression was used to relate the numbers of children admitted and the number of deaths to the catchment population at different distances from Teule. Logistic regression was used to obtain odds ratios (OR) and 95% confidence intervals (95% CI) for individual risk factors on mortality for children admitted to Teule hospital.

### Ethics

The study was approved by the Ethics Committees of the National Institute for Medical Research, Tanzania, and the London School of Hygiene and Tropical Medicine, UK. Witten informed consent to participate was obtained from the parent or guardian of each child in the study.

## Results

### Overview and characteristics of children in the study

Of 3639 children in the original study, 286 were excluded as they were over the age of 59 months and 242 were excluded due to residence outside the catchment area, leaving 3111 children included in the analysis of whom 143 (4.6%) died. Among the children admitted to hospital, males outnumbered females and the proportion of males increased with travel time from the hospital, as did the proportion of mothers who had not completed primary education. There was also a trend towards increased pre-admission duration of illness and a shorter time between admission and death with increasing travel time to hospital. (Table 1). A total of 258 (8.3%) children had been referred from another health facility.

### Admission and death rates by travel time to hospital

**Figure 2:**
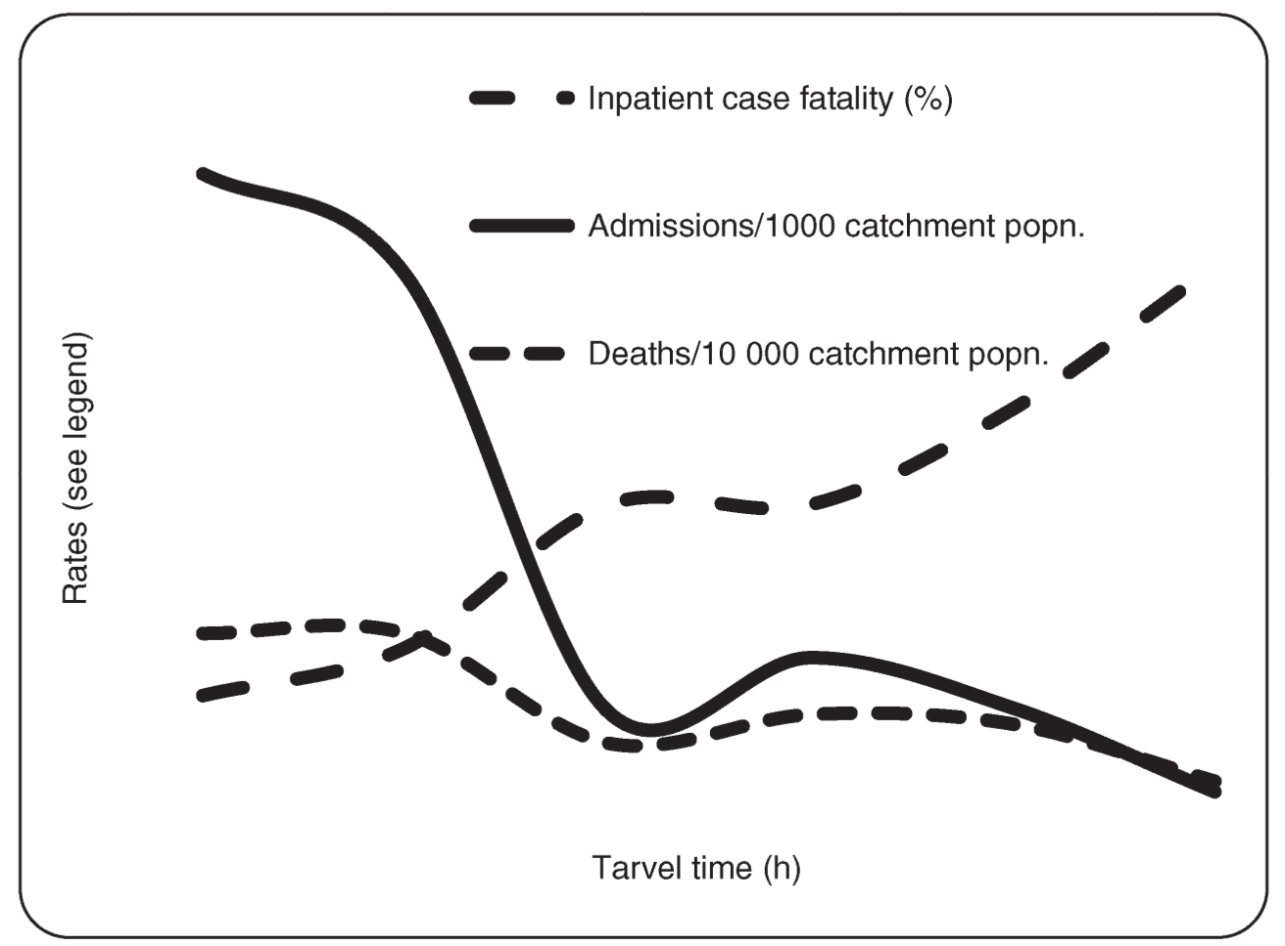
Admissions and deaths under the age of 59 months shown as the case fatality rate or per catchment population.

Overall 2307/3111 (74%) of admissions came from villages located at <3 h travel time from the hospital while these villages included only 1 8519/50 359 (36.8%) of the catchment population. Admission rates declined from 125/1000 under-5 catchment population living in villages located at <3 h travel time, to 25/1000 in villages located at 3 h or more travel time to the hospital (Table 2 and Figure 2).

**Figure 3:**
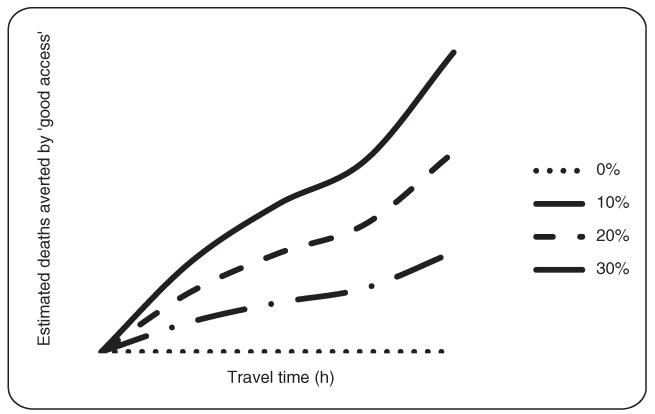
Estimated cumulative deaths averted among the 50 359 children in the catchment population if children living ≥3 h from the hospital had been admitted at the same rate and mortality risk as children admitted <3 h away.

Figure 3 shows the projected deaths that might have been averted if admission and death rates that were observed for children coming from villages <3 h travel time from the hospital had applied equally to the whole catchment population. As the benefit attributable to inpatient compared to outpatient care is not known, a sensitivity analysis was used to determine deaths that could have been averted by living at <3 h travel time to hospital using estimates of hospital/non-hospital benefit between 0% and 30%. If hospital care compared to outpatient care had reduced mortality in those admitted by 30%, then child mortality due to severe febrile illness in the catchment population would have been reduced by 45/440 (10.3%).

Poisson’s regression analysis of inpatient deaths per catchment population showed that the rate of inpatient mortality/1000 catchment population declined by 21.4% per 1 h of travel time from the district hospital (*P* < 0.001) (Table 2). Assuming that child mortality was uniformly distributed in the catchment population, an estimated 21.4% additional deaths occurred without the benefit of inpatient care prior to death per hour of travel to the hospital. The proximity of an alternative primary care facility did not have a significant effect on this result (*P* = 0.64).

### Admission and death rates by individual factors

Risk factors associated with inpatient mortality are shown in Table 3. There was a strong association between mortality and travel time such that the adjusted odds of mortality among children admitted from villages that were more than 6 h travel time from the hospital was 4.31 (1.96–9.53) compared to children admitted from villages located <2 h away (Table 3). The proportion of males among admissions arriving from villages <3 h travel time away was slightly higher than females (1230/2307, 53.3%), and among these admissions, there were 39/1230 (3.2%) and 40/1077 (3.7%) deaths among males and females, respectively (*P* = 0.47). However, these differences were greater among admissions from villages that were 3 h or more distant from the hospital where 434/804 (54%) were male and the case fatality with 28/462 (6.1%) and 36/342 (10.5%) among males and females, respectively (*P* = 0.021).

### Travel time and adjusted distance

**Figure 4:**
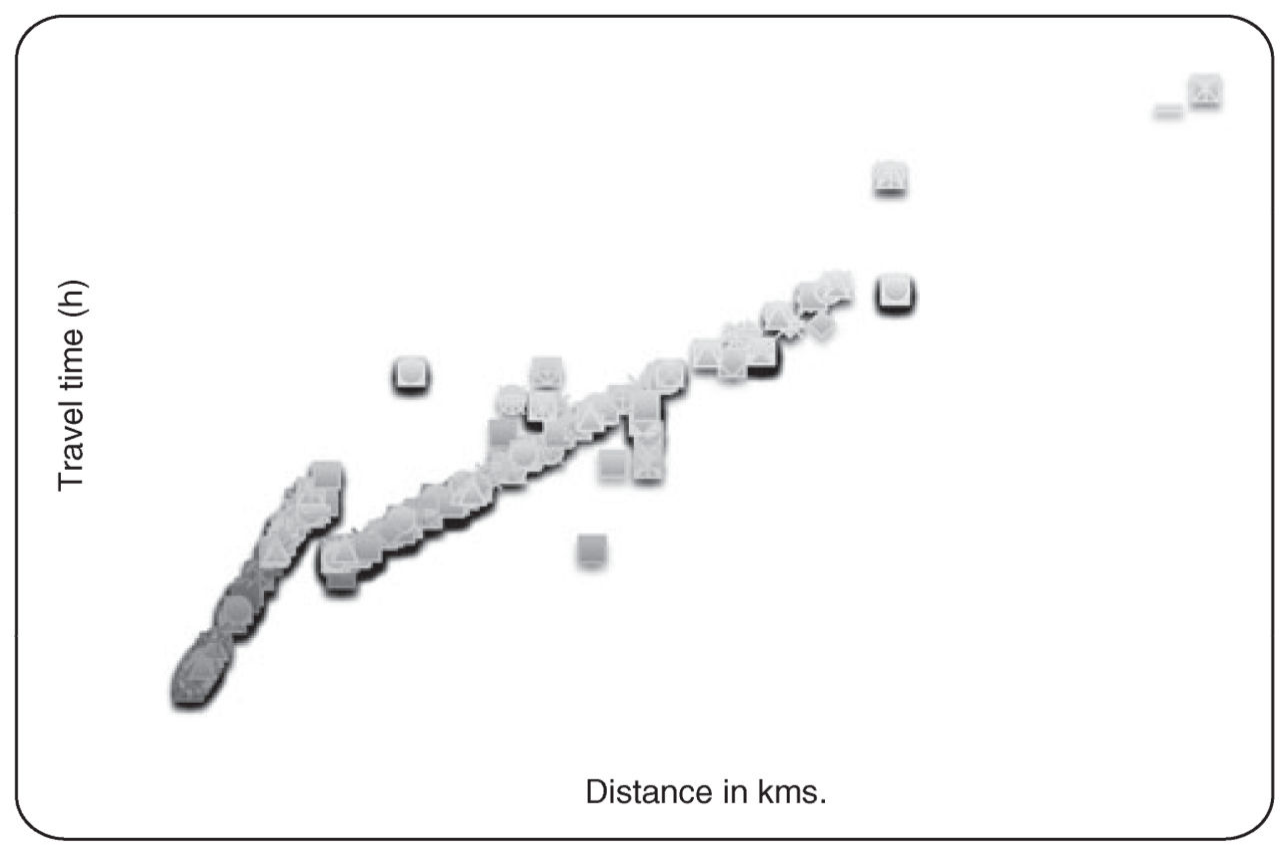
Scatter plot of estimated travel time against adjusted distance to Teule Hospital from villages in the catchment population.

The relationship between travel time and distance was generally linear with some variation for those who were presumed to walk (residence <7 km from hospital) compared to those living between 7 and 12 km distant who were judged to have used a combination of walking and motorised transport (Figure 4).

## Discussion

Our results suggest that the association between inpatient mortality in children and the distance travelled to hospital is largely the product of an excess of low-risk admissions from villages relatively close to the hospital. However, for children living in more distant villages, we have also demonstrated an unmet need for hospital admission where twice as many childhood deaths occurred without the benefit of inpatient care for populations living more than 3 h or more from hospital as occurred in populations living <3 h away.

Translating these findings to likely mortality effects in the catchment population is difficult for a number of reasons: accurately defining the catchment population, taking account of alternative health care providers and the fact that there is little evidence as to the influence of hospital care compared to home-based care in sick children. The latter gap in knowledge is remarkable given the large investment that is made in hospital care. The mortality benefit of hospital care might be estimated from considering the effect of treatments such as intravenous therapy, oxygen or blood that are known to be available in hospital but not in the community. However, this has also proved to be difficult; a Delphi consultation with a panel of well-recognised experts in childhood febrile illness in resource-poor countries found a very low level of agreement on the contribution of medical care in reducing mortality due to common childhood diseases. (Lubell *et al.* 2011).

In the absence of alternative evidence, we have used a sensitivity analysis of ‘hospital benefit’ that varied from ‘no benefit’ to ‘reducing mortality by 30%’. The choice of this range was based on the differential mortality of patients given better treatment compared to ‘routine’ treatment in clinical trials at the same hospital where it seems conservative to estimate a maximum 30% mortality differential. and this then would suggest that approximately 10% of deaths in the catchment population of the study hospital might not have occurred if more distant populations had been provided with the same level of hospital access as those living nearby (Dondorp *et al.* 2010; Maitland *et al.* 2011).

There also appeared to be a gender bias in seeking care; the increasing proportion of male children with distance suggests that, where additional resources are required to seek care, boys are increasingly favoured. The effect did not appear to be the result in any difference in need for hospital admission as female mortality was actually higher than male. At least one other hospital-based study has observed a similar gender bias (Schellenberg *et al.* 1999). The fact that this effect generally requires large numbers to reach statistical significance may be one factor that has prevented this effect from attracting greater attention. Tanzania does not appear to be atypical of resource-poor countries in its gender bias practices, and following more than two decades of socialism may be less prone to gender bias than some other resource-poor countries.

What can be expected in the future? There is evidence of declining child mortality but no studies that we are aware of that have determined in what groups this decline has occurred. It seems likely that the decline has occurred where access to care and improved living standards that accompany economic developments that the decline may well be greatest in populations with good access to care and health services generally (Bernard *et al.* 2009). This raises the possibility that child mortality may become increasingly concentrated in poor and remote communities.

### Limitations to the study

The study rests on a number of estimates and assumptions that have been specified, and these are inevitably subject to uncertainty and potential error. The study excluded admissions of very young infants (<2 months of age), children over the age of 5 years and admissions for non-febrile or chronic illness such as elective surgery, malignancy. In addition, night-time and some weekend admissions were not included. Admissions for surgery or malignancy were uncommon but admissions outside normal working hours may have introduced bias, and it is difficult to predict the direction of this as distant populations may seek care in some desperation, but transport was often not available from distant villages at night.

The observed association between travel time and mortality could, at least in part, be influenced by a variety of potential confounders such as excessive use of traditional medicines or greater exposure to disease in different areas of the district. Unfortunately data on these were not collected. Fewer than one in ten children in the study had been referred from another facility, but the proportion of these increased with increasing travel time from the hospital. However, without data on referrals that were made from peripheral clinics, it is difficult to draw conclusions.

It is possible that there were preferences in the local population in choosing hospital services that could invalidate our estimate of the catchment population although our observations of increasing case fatality, time to death and pre-admission drug use all support a conclusion that distant populations were seeking treatment later and in more desperation than proximal populations. We were unable to quantify the possible use of smaller or private inpatient care facilities although our model did not demonstrate a significant effect of alternative providers on hospital usage.

Access to health care is multidimensional while in this study the only measure of socio-economic status was self-reported education of the mother, and this has limited our ability to take into account factors such as household wealth and preference for traditional medicine. The influence of socio-economic factors on child mortality in this study remains uncertain but is likely to increase the measure of unmet need for hospital care for sick children due to poor access as less educated and more remote populations are generally exposed to a higher burden of illness and death irrespective of access to care (Frankenberg 1995).

## Conclusions

The use of hospital services and inpatient case fatality rates are strongly related to travel times to hospital, and this was most pronounced within 2 h of travel to the hospital. We estimate that approximately 20% more fatal illnesses occurred without the benefit of inpatient care for every 1-h increase in travel time to hospital, and this is likely to apply to many rural areas of Africa. How this is likely to affect child mortality depends on the impact of inpatient care on case fatality rates about which surprisingly little is known. The findings of this study suggest that in rural areas of Africa provision of public transport, better roads or promotion of alternative affordable means of transport are likely to improve child survival in addition to having more direct economic benefits.

## Figures and Tables

**Table 1:** Characteristics of children included in the study by travel time to hospital

Travel time (h)	<2 h	2–2.9 h	3–3.9 h	4–4.9 h	5–5.9 h	6+ h
Children admitted	780	1527	245	317	159	83
% Male	52	54	57	56	60	59
% <12 months of age	32	29	36	32	45	29
None or incomplete education of mother, %	7	11	14	18	26	26
Referred from another facility, %	1	3	5	4	5	17
Medication in 48 h prior to admission, %	78	82	87	82	88	90
Pre-admission days ill	3.7	4.1	4.5	4.2	4.6	4.8
Duration of admission (days)	3.2	3.3	3.7	3.5	3.4	3.9
Days to death (*n* = 143)	4.0	1.6	2.0	1.3	0.8	1.3
% *Plasmodium falciparum* positive	52	67	58	59	67	54
Mean Hb (g/dl)	8.7	7.9	7.3	7.1	6.6	6.1
Invasive bacterial disease (%)	7	9	14	11	11	20
Case fatality rate (%)	3	4	7	7	9	12

**Table 2:** Hospital admissions, case fatality and deaths by catchment population of the study hospital by travel time from village of residence

Travel time (h)	Admissions	Deaths	CFR% (95% CI)	Population	Admissions/1000 p-years (95% CI)	Deaths/1000 p-years (95% CI)
0–2	780	24	3.1 (2.0–4.6)	5601	138 (129–148)	4.3 (2.7–6.4)
2–2.9	1527	55	3.6 (2.7–4.6)	12918	117 (111–123)	4.3 (3.1–5.5)
3–3.9	245	18	7.5 (4.5–11.5)	9193	26 (23–30)	2.0 (1.2–3.1)
4–4.9	317	22	7.0 (4.4–10.4)	8371	38 (34–42	2.6 (1.6–4.0)
5–5.9	159	14	8.9 (4.9–14.4)	5798	27 (23–32)	2.4 (1.3–4.1)
6+	83	10	12.2 (6.0–21.3)	8478	9.7 (7.7–12.0)	1.2 (0.5–2.2)
All	3111	143	4.6 (3.9–5.4)	50359	61 (59–63)	2.8 (2.4–3.3)

**Table 3:** Odds ratios for inpatient mortality by travel time and other socio demographic characteristics

	Crude odds (95% CI)	*P*	Adjusted odds (95% CI)	*P*
Travel time				
0–2 h	1		1	
2–3 h	1.17 (0.72–1.92)	0.512	1.12 (0.69–1.84)	0.646
3–4 h	2.50 (1.33–4.68)	0.004	2.23 (1.17–4.27)	0.015
4–5 h	2.35 (1.30–4.25)	0.005	2.29 (1.25–4.18)	0.007
5–6 h	3.04 (1.54–6.02)	0.001	2.7 (1.34–5.45)	0.006
6+ h	4.32 (1.99–9.37)	<0.001	4.31 (1.96–9.53)	<0.001
Age group				
2–11 months	1		1	
12–23 months	0.56 (0.40–0.78)	0.001	0.57 (0.40–0.80)	0.001
24–59 months	0.60 (0.98–4.51)	0.621	0.58 (0.01–4.41)	0.595
Female	1.37 (0.98–1.92)	0.065	1.36 (0.96–1.91)	0.081
Mother completed primary education	0.82 (0.66–1.01)	0.064	0.92 (0.73–1.14)	0.439
No. days ill pre-admission	1.01 (0.96–1.05)	0.562	1.01 (0.96–1.04)	0.857
Any medication within 48 h of admission	1.56 (0.94–2.57)	0.084	1.35 (0.81–2.24)	0.253
